# The Novel Roles of Connexin Channels and Tunneling Nanotubes in Cancer Pathogenesis

**DOI:** 10.3390/ijms19051270

**Published:** 2018-04-24

**Authors:** Silvana Valdebenito, Emil Lou, John Baldoni, George Okafo, Eliseo Eugenin

**Affiliations:** 1 Public Health Research Institute (PHRI), Newark, NJ 07103, USA; sv505@njms.rutgers.edu; 2 Department of Microbiology, Biochemistry and Molecular Genetics, Rutgers New Jersey Medical School, Rutgers the State University of NJ, Newark, NJ 07103, USA; 3 Department of Medicine, Division of Hematology, Oncology and Transplantation, University of Minnesota, Minneapolis, MN 55455, USA; emil-lou@umn.edu; 4 GlaxoSmithKline, In-Silico Drug Discovery Unit, 1250 South Collegeville Road, Collegeville, PA 19426, USA; John.M.Baldoni@gsk.com; 5 GlaxoSmithKline, In-Silico Drug Discovery Unit, Stevenage SG1 2NY, UK; George.N.Okafo@gsk.com

**Keywords:** intercellular communication, gap junctions, hemichannels, tumor microtubes, glioblastoma

## Abstract

Neoplastic growth and cellular differentiation are critical hallmarks of tumor development. It is well established that cell-to-cell communication between tumor cells and “normal” surrounding cells regulates tumor differentiation and proliferation, aggressiveness, and resistance to treatment. Nevertheless, the mechanisms that result in tumor growth and spread as well as the adaptation of healthy surrounding cells to the tumor environment are poorly understood. A major component of these communication systems is composed of connexin (Cx)-containing channels including gap junctions (GJs), tunneling nanotubes (TNTs), and hemichannels (HCs). There are hundreds of reports about the role of Cx-containing channels in the pathogenesis of cancer, and most of them demonstrate a downregulation of these proteins. Nonetheless, new data demonstrate that a localized communication via Cx-containing GJs, HCs, and TNTs plays a key role in tumor growth, differentiation, and resistance to therapies. Moreover, the type and downstream effects of signals communicated between the different populations of tumor cells are still unknown. However, new approaches such as artificial intelligence (AI) and machine learning (ML) could provide new insights into these signals communicated between connected cells. We propose that the identification and characterization of these new communication systems and their associated signaling could provide new targets to prevent or reduce the devastating consequences of cancer.

## 1. Introduction

According to the American Cancer Society, 1,685,210 new cancer cases are detected and 595,690 cancer deaths are projected to occur in the USA each year, making this disease the second highest cause of death [1,2]. Even though there are several types of cancers that are potentially curable or have favored outcomes, there are numerous others—pancreatic carcinoma and glioblastoma (GB) among them—that are almost always incurable and have particularly poor prognoses, even with the most aggressive therapeutic interventions [3,4,5,6,7]. GB in particular is the most aggressive diffuse glioma of astrocytic lineage and remains incurable with a mean survival of 15 months after detection [6,8]. Thus, there is an urgent need to identify new mechanisms to design more effective and different therapeutic interventions against this cancer.

It is widely accepted that cancer is a genetic disease in which multiple genomic alterations result in the uncontrolled growth, dedifferentiation, and invasion of healthy tissues. However, recent advances in DNA sequencing, epigenetics, microscopy and radiologic imaging, proteomics, lipidomics, and metabolomics (OMICS) have all resulted in the identification of new pathways involved in carcinogenesis. Currently, there are large amounts of high-quality genomics and protein databases for a variety of cancers at different differentiation stages which clearly underscore the importance of cell-to-cell communication in tumor evolution and prognosis (see [9]). At this time, one of these novel approaches is artificial intelligence (AI). These in silico approaches are able to handle large datasets from multiple areas of research, including patient information, disease progression, treatments, genetics, pathology, and OMICS, to identify new affected pathways and potential drug targets. However, this exciting approach is still in its infancy [10].

## 2. Artificial Intelligence and Machine Learning: New Tools to Identify Drug Targets and Common Pathways of Disease

**Figure 1:**
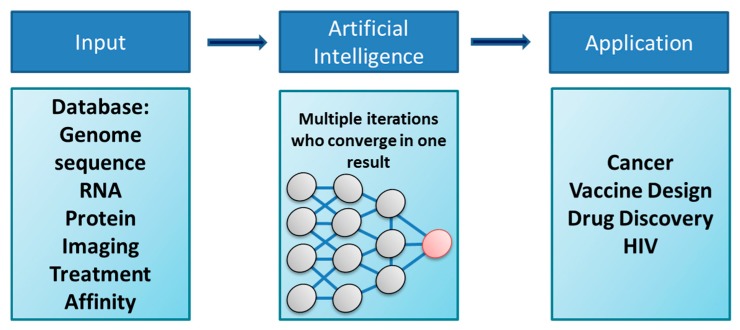
Cartoon denoting the main aspects of deep learning algorithms and artificial intelligence (AI) in biology. As described in the text, critical points include the input information, the features selected for analysis, the training of the AI, the numbers of layers or variables or classifiers, as well as the desired output. The unbiased connection between the different clusters or layers of the AI system will provide the best predictors for associated gene/mRNA/protein for a particular biomarker. If this biomarker is unknown, the clusters can select the dysregulated pathways or better treatments based on the input information. Thus, the possibilities are endless.

Artificial intelligence and machine learning (ML) are emerging in healthcare to help answer key questions in tumor biology and cell-to-cell communication [11]. The existing and emerging vast data on tumor biology and associated OMICS require integration at a multi-dimensional scale [12]. In non-healthcare sectors, AI methods have been developed to analyze multi-dimensional data to test hypotheses [13]. AI algorithms are emerging and can be used to interrogate biological mechanisms and dysregulated pathways associated with cancer phenotypes and cell-to-cell communication with intent to improve cancer treatments [11]. In this instance, AI inputs datasets (also called features or variables), processing them via classification algorithms (common examples include artificial neural networks, correlative analysis, and generative adversarial networks) to generate outputs (also called predictions) or hypotheses to be tested [14] (exemplified in Figure 1).

In the context of cell-to-cell communication in cancer, these variables could include normal versus diseased patient-derived cell clinical, treatment-based, metabololomic, proteomic, and transcriptomic data (any type of OMICs data) and genomic information; up- or downregulated pathways associated with different types or outputs of cancers; the response of patient-specific phenotypes to drug therapy at the molecular level; the modulation of metabolic pathways within cells; the transference of genetic information from cell-to-cell within a tumor; and the evolution of the tumor microenvironment during the progression of the disease in the patient, for example. Each of these variables can be considered a cluster, as indicated in Figure 1.

Growing in vitro evidence suggests that tunneling nanotubes (TNTs), hemichannels (HCs), and gap junctions (GJs) play a role in the tumor microenvironment [15]. The outputs of these (and other) analyses are highly interrelated and generate multivariate associated information, such as potential drug targets, signaling and biochemical pathways with intersection nodes, metabolic signatures, biochemical biomarkers of disease at a point in time, and increasingly image-based evidence of how the biology evolves in real time. The utility and relevance of ML is highly dependent on the following key factors: access to high-quality, well-curated, labeled datasets, which are required to both train the algorithm and allow it to rapidly and accurately distinguish between features and recognize patterns; access to high-performance computing capability, preferably cloud-based for the speed and computing power necessary to process large volumes of multivariate data; and the incorporation of disease domain expertise to provide scientific context to ensure the algorithm makes logical correlations (see flow in Figure 1). Correlative analytical algorithms (CAAs) are a good example of ML and have been applied in cancer biology [11]. CAAs use large quantities of text-based data sources to identify links and patterns amongst different datasets and differentiate the information into many hypotheses for further scrutiny by the domain expert. Also, these algorithms can help to elucidate intracellular mechanisms i.e., to connect the dots among gene interactions inside cellular networks longitudinally with time (so-called cause-and-effect cellular networks) as well as cell-to-cell interactions and the subsequent signaling pathways involved in TNTs, HCs, and GJs communication. This AI approach has been used in patients with somatic mutations in lung cancer to better classify the type of tumors using four large datasets and with focus on kRAS and epidermal growth factor receptor (EGFR), enabling a 75% prediction of mutations associated with these genes [16]. A similar correlative AI approach was used to build breast cancer models by combining transcriptomic and genomic signatures from healthy and breast cancer subjects with information from known patient medical histories to identify patterns or hot spots in their molecular signatures, leading to the discovery of biomarkers, drug targets, and subtypes of breast cancer [17]. Thus, the analysis of multiple variables and communication networks as well as particular communication systems (connexin (Cx)-mediated systems) can provide a unique platform to identify pathways and communication systems altered during the pathogenesis of cancer, including localized gap junctional and HC communication.

## 3. Connexins and Cancer

A critical feature observed in all OMICS studies is the lack of or reduced communication between cancer cells and non-cancer surrounding cells [18,19,20]. However, it only recently became clear that cancer cells regulate cell-to-cell communication to promote cancer cell invasion and spread to local and distant sites using GJs, HCs, and TNTs [15,21,22,23]. GJ channels are formed by two HCs, and each HC is formed of connexin (Cx) hexamers enabling the communication with the cytoplasm of neighboring cells [24,25,26,27]. Connexin-containing HCs can be formed by one (homomeric connexins) or several (heteromeric) types of Cxs. GJs can be docked by two identical (homotypic) or different (heterotypic) subunits of HCs. These multiple combinations produce channels that differ in their biophysical properties and permeability [28,29]. The large internal diameter of the pore of these channels is around 12 Å. The pore enables ions and intracellular messengers less than 1.2 kDa, including IP_3_, calcium, cyclic nucleotides, metabolites, toxic molecules, neurotransmitters, viral peptides, and electrical signals, to diffuse between connected cells [24,25,26,27]. Through the diffusion of these second messengers among connected cells, GJs coordinate physiological functions including cell proliferation, differentiation, and homeostasis maintenance [26].

In general, it is well accepted that the loss of Cx expression and downregulation of GJ communication is associated with cancer progression [30,31,32,33], and it is hypothesized that this lack of communication enables pre-cancerous cells to proliferate without the cell-to-cell control of neighboring cells. There are several outstanding reviews and primary manuscripts describing the downregulation of Cxs in cancer [33,34,35]. Thus, we will not be describing these well-accepted mechanisms. Here, we will describe the new roles of localized GJs, HCs, and TNTs in tumor progression and spread.

## 4. Mechanisms of Cancer Initiation and Spread: Potential Implications of the Intercellular Transfer of Genetic Alterations

The pathogenesis of cancer is complex, resulting from a series of missteps at the genetic and metabolic levels. It is widely accepted that cancer is initiated by alterations in the cell genome (nuclear DNA, nDNA) such as mutations, deletions, methylation, or miss-orientations, which result in uncontrolled proliferation and immune evasion [36]. Under healthy conditions, natural killer (NK) cells have the capability of recognizing and killing tumor cells without the requirement of prior antigen exposure [37]. However, if the rate of cancer cell proliferation is high or immune activation is compromised, tumor growth becomes uncontrollable, resulting in carcinogenesis. Currently, immunotherapeutic drugs are under investigation in clinical trials, and some are already approved by the U.S. Food and Drug Administration (FDA), with the goal of reprogramming the immune system to recognize and attack cancer cells as foreign rather than as self-entities. The ability of this class of drugs to work by affecting mechanisms of cancer initiation and spread at the earliest stages is still under active investigation.

It is well accepted that a major cause of carcinogenesis is the generation of irreversible mutations or DNA alterations that create “cancer-like” or “cancer stem” cells. However, the mechanism of how these cells are generated is poorly understood. It has been proposed that a single cell containing a pro-carcinogenic mutation proliferates uncontrollably to create a tumor. A different, but not exclusive, hypothesis posits that this irreversible genetic aberration(s) and associated metabolic/genetic behavior can be spread into neighboring cells by GJs, HCs, and TNTs, as well as exosomes.

Currently, at least four sources of DNA mutations in normal cells are known: quantum effects base pairing, mutations due to errors in DNA polymerase activity, hydrolytic deamination of bases, and damage induced by endogenously produced reactive oxygen species or other metabolites [2]. The damage in nDNA can be repaired by six pathways: direct reversal of the mutation, nucleotide excision, base excision, mismatch and recombinational repair, translesion synthesis, and chemical reactions such as hydrolysis and methylations [38,39]. Some stochastic damage in the DNA is regulated by checkpoint pathways, which typically involve proteins such as cyclin-dependent protein kinases (CDK) and tumor suppressors, such as the retinoblastoma protein and p53 [40]. Usually, the repair of nDNA is dependent on two factors: the type of nDNA lesion that needs to be repaired and the cell cycle stage during which the repair takes place [41]. Depending on the severity of the nDNA damage, the efficacy of checkpoint pathways can decrease considerably and their dysfunction can result in cell death or cell cycle reprogramming, increasing the likelihood of carcinogenesis. However, the amplification mechanism for compromised DNA is unknown and cannot be explained by clonal proliferation. Thus, a different mechanism of amplification is necessary. We propose that TNTs, GJs, and HCs could provide an alternative mechanism of lateral DNA diffusion.

An example of lateral DNA transfer has been observed in mitochondrial DNA (mtDNA) by a mechanism that might involve GJs, HCs, and TNTs. Over the past decade, the study of mitochondria has mainly focused on their role as a bioenergetics and biosynthetic factory through the synthesis of adenosine triphosphate (ATP). However, the important role of mtDNA in carcinogenesis is becoming increasingly clear [42,43,44,45,46,47,48]. mtDNA is dependent upon many nuclear proteins for transcription, translation, replication, and repair. mtDNA is also more mutable and evolves 5–10 times faster than nDNA [49,50]. The mean rate of divergence over the whole mtDNA molecule is ~2% per 106 years [51]. This can be explained by three possible causes [52]. Firstly, because mtDNA lacks histones (responsible for the regular packaging of nuclear DNA into nucleosomes) and chromatin, making it more susceptible to oxidation by free radicals [52,53,54,55]. Secondly, mitochondria have an inefficient system of DNA repair [46,56]. Thirdly, mtDNA is subject to continued exposure to reactive oxygen species (ROS) because of the close proximity of mtDNA to the electron transport chain. Indeed, ROS and ROS-mediated DNA oxidation are known to participate not only in the initiation but also in the propagation of cancer [52,57].

Recently, several groups demonstrated the exchange of mitochondria via TNTs [15,58,59,60,61,62,63], illuminating a way in which mutated mtDNA or healthy mtDNA could be shared between cells. Furthermore, the selective formation of TNTs (open-ended and enclosed with connexins at the tip) between tumor cells and healthy cells facilitates the spread of several mRNAs, microRNAs, proteins (including oncogenes), and second messengers from the tumor cell to the target cell to change its metabolism and become “cancer-like” despite the absence of the DNA alterations that characterize the original tumor. Based on these observations, our hypothesis is that TNTs and GJs between tumor cells and between tumor cells and healthy cells promote the exchange of material (e.g., mutated mtDNA) to better adapt to changes in tumor metabolism and dedifferentiation events promoted by the tumor. In addition, the opening of HCs and their release of intracellular metabolites could change the metabolic/inflammatory status of neighboring cells by the local release of ATP and onco-metabolites.

Our hypothesis is that carcinogenesis can be favored by original mutations in mtDNA that can be transferred via TNTs and affect the nDNA structure, replication, and repair. Around 99% of the mitochondrial proteins are encoded, regulated, transcribed, and their genes replicated in the nucleus, which further underscores the importance of the intracellular communication between the mitochondria and nucleus. This communication is known as mitochondria-to-nucleus “retrograde signaling” [64,65,66]. Typically, each cell contains 10^3^–10^4^ copies of mtDNA, which can replicate independently of nDNA [66,67]. Most mitochondrial proteins are encoded by nDNA and are translated in the cytosol prior to their active transportation into the mitochondria, which retain a small 16 Kb mtDNA genome that encodes tRNAs, rRNAs, and proteins essential for metabolic respiration (a limited number of the electron transfer chain (ETC) proteins (complex I: (ND1–ND6 and ND4L), complex III: (apocytochrome b subunit), complex IV: (COXI, COXII, and COXIII), and ATP synthetase (ATPase6 and ATPase8) [68]. Thus, the communication between the nuclear and mitochondrial DNA is extremely dependent on the nuclear-mitochondria communication. We propose that the exchange of compromised mtDNA will affect the expression of nuclear genes in the targets cells enabling a better adaptation of carcinogenesis and the associated metabolic changes observed in healthy cells surrounding cancer cells.

Only recently it has become evident that mitochondria onco-metabolites such as α-ketoglutarate (KG), d-2-hydroxyglutarate, and fumarate work as epigenetic modulators, especially in the pathogenesis of GB [69,70,71]. For example, KG is a Krebs cycle metabolite that regulates anabolic and catabolic at the citrate cycle (TCA) products and substrates [72]. KG is an obligatory co-substrate for 2-oxoglutarate-dependent dioxygenases (2-OGDDs) involving hydroxylation reactions on various types of substrates including proteins, nucleic acids, lipids, and metabolic intermediates [73,74]. As a substrate of hydroxylases, KG exerts an impact on prolyl/aspartyl/lysyl hydroxylations, which in turn regulate the stability of the hypoxia-inducible factor (HIF)-1 and collagen synthesis, both important factors in cancer development. In addition, prolyl hydroxylases (PHD1–3) influence the function of HIF-1 [75,76,77], an important transcription factor in cancer development and progression [78]. Furthermore, KG binds and regulates G-protein function, because it is a ligand for G-protein-coupled receptor GPR99/GPR80, which acts exclusively through a Gq/11-mediated pathway [79]. Signaling through this pathway mobilizes intracellular Ca^2+^ (via the activation of phospholipase C), which acts as a diffusible second messenger regulating a wide range of vital cell functions, including cellular metabolism and growth, cell division and differentiation, and carcinogenesis [80]. Therefore, KG can also function as a signaling molecule. Our hypothesis is that GJs, HCs, and TNTs help the exchange of compromised DNA and onco-metabolites to accelerate tumor growth and the adaptation of neighboring tissues to the tumor.

As described above, each cell contains 10^3^–10^4^ copies of mtDNA, which can replicate independently of nDNA [66,67]. Thus, it is unclear how mtDNA homoplasmic mutations (cells within a tumor that carry the same mtDNA mutation) can be transmitted and maintained independently of nDNA replication or cellular proliferation [53,81]. mtDNA mutations in nicotinamide adenine dinucleotide (NADH) dehydrogenase (respiratory complex I) subunit 5 gene (*ND5*) might play an important role in the early stage of carcinogenesis, possibly through increased ROS generation and apoptosis [82,83]. Previous studies showed that the mtDNA mutations G13997A and 13885insC in the gene encoding NADH dehydrogenase subunit 6 gene (*ND6*) have a compromised respiratory complex I and subsequently overproduce ROS, increasing metastatic potential [84]. Mutations in mtDNA could also alter the structural conformation of this biomolecule. For instance, mitochondrial D-loop alterations may constitute inherent risk factors for cancer development [85]. In general, somatic mutations in mtDNA and alterations in mitochondrial function are associated with the initiation of different tumors [83], and affect such different tissues as bladder [86,87,88], breast [89,90,91], colorectal [92,93,94], head and neck [95,96,97,98], brain [99,100,101], thyroid [102], kidney [103,104], liver [105], lung [106], and stomach tissues [107], as well as being found in leukemia and lymphoma cancers [108]. However, their role in carcinogenesis, tumor growth, and spread is not completely understood. Based on the well-established finding that particular mutations in mtDNA are highly associated with specific types of tumors, we hypothesize that TNTs and GJs that connect among tumor cells and connect tumor cells to healthy cells promote the exchange of mutated mtDNA in order to permit changes in tumor metabolism and dedifferentiation events that sustain and favor further cancer growth.

**Figure 2:**
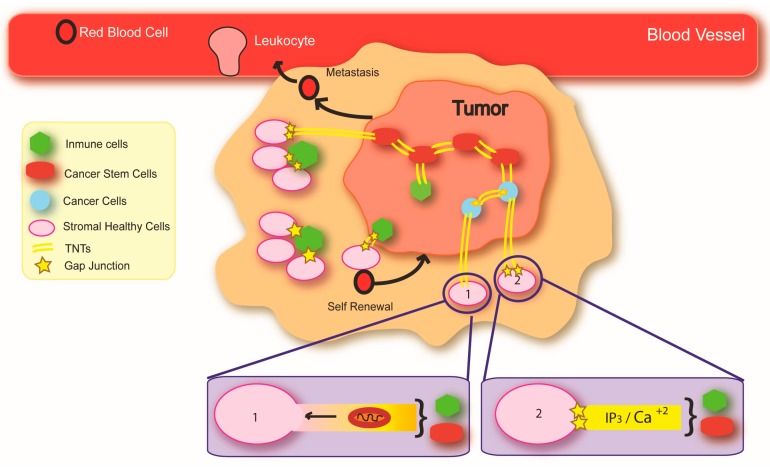
Representation of the potential role of gap junctions (GJs), hemichannels (HCs), and tunneling nanotubes (TNTs) in carcinogenesis. GJs and TNTs are expressed in a localized manner to help the tumor to invade neighboring tissues. Tumor cells (stem cells and cancer cells) communicate between them and surrounding cells (stromal healthy cells, immune cells) through TNTs and GJs, transferring organelles and metabolic agents from cancer stem cells and immune cells to stromal healthy cells. (1) An open-ended TNT allows the exchange of organelles (mitochondria), vesicles, and small molecules between the connected cells. (2) The open-ended TNT contains Cx protrusions that allow the exchange of small molecules, such as Ca^2+^ or inositol triphosphate (IP_3_), between connected cells.

Interestingly, when compared to cancer, human immunodeficiency virus (HIV) infection has shown similar metabolic mechanisms of pathogenesis. For instance, both diseases are characterized by DNA (nuclear and mitochondrial) repair issues, external DNA insertion, viral use of host genes, and activation of oncogenes [109,110,111,112,113,114,115]. In agreement, several viral infections are highly associated with cancer development; for example, in almost all cancer biopsies of cervical cancer cells, DNA expression of specific viral Human Papillomaviruses (HPVs) genes (such as E6 and E7) have been found [116]. In HIV, the virus adapts in order to use Cxs-containing channels to maintain communication with uninfected cells to support the survival and spread of viral infection [15,117]. These similar mechanisms could explain the spike in cancers observed in the HIV-infected population [109,110,111,112,113,114,115]. Our hypothesis is that both diseases employ Cxs, GJs, HCs, and TNTs in a similar fashion to spread toxicity, metabolic changes, and cancer phenotypic signaling, as well as to provide resistance to hypoxia and several anti-cancer/HIV treatments (see Figure 2). In both cancer and HIV, tumor growth and HIV-associated damage is highly localized to neurovascular areas [118,119,120]. This localized damage is characterized by a local blood-brain barrier (BBB), vascular or mitochondrial compromise, dysregulation of GJs, and increased dependency on unusual sources of energy such as glutamate/glutamine [121,122,123]. Our laboratory has shown that HIV infection of microglia/macrophages or astrocytes results in the upregulation of Connexin43 (Cx43), the key protein of GJs in these cell types, as well as TNTs, which helps promote the spread of pro-inflammatory signals from infected to uninfected areas [15,117]. Thus, we propose that HIV as well as cancer cells cause and propagate damage by “hijacking” GJs, HCs, and TNTs to spread toxic signals that compromise neighboring cells (Figure 2).

## 5. Cancer and Metabolic Compromise: Focus on Central Nervous System Malignancies

A major requisite for tumor growth is the supply of sufficient nutrients and oxygen via blood vessels. Therefore, a critical event in carcinogenesis is the adaptation of cancer cells and healthy cells around the tumor (vascular and non-vascular cells) to the changing metabolic conditions [124]. Indeed, brain tumor stem cells often localize and associate with perivascular regions to acquire nutrients and spread into other tissues [125]. Furthermore, while healthy cells mostly depend on energy production via the activation of the oxidative phosphorylation (OXPHOS) system, it has been accepted that cancer cells mostly use glycolysis and lactate production as well as particular amino acids under hypoxic conditions [126]. Cx43-containing channels are the pathway to discharge lactate to promote adenocarcinoma growth [127]. Our preliminary data indicate that HIV reservoirs and glioblastoma cells have similar mtDNA modifications, resulting in better adaptation to low oxygen levels or hypoxia (Figure 2). The spread of these mtDNA modifications was in fact mostly mediated by TNTs, suggesting that both TNTs and GJs contribute to the spread of these mutations and metabolites to aid the adaptation of the tumor.

It is accepted that general decrease in cell-to-cell communication, Cx expression, and GJ and TNT communication is closely associated with tumor progression [9,33]. However, new data indicate that junctional proteins are concentrated in tumor microtubes (TMs), a variant of TNTs that are micro-sized in width and length compared to most reported forms of TNTs, as studied in an in vivo animal model of GB [128]. TNTs/TMs in gliomas use several developmental proteins to establish contacts with healthy cells and spread cancer as well as to facilitate treatment resistance [129]. We recently reported our finding that TNTs contain GJs and HCs at the tips of TNT processes; in this setting, GJs and perhaps HCs enhance cell-to-cell interaction for infection and viral spread [130]. In cancer, stem cells express Cx46 and form the functional channels required for tumorigenesis; blocking these channels results in decreased proliferation, self-renewal, and tumor formation [30,131], suggesting that targeting the low expression of Cxs channels and TNTs could be used to target GB and maybe other types of cancers.

Recently, we demonstrated that there are at least two different kinds of TNTs: one type containing GJs at the end of the process and another fused with the recipient cell, enabling the exchange of vesicles and organelles between connected cells [15,132]. TNTs proliferate during embryonic development and under pathological conditions, especially cancer [133,134]. TNT formation has been observed in tissue culture in epithelial, endothelial, mesenchymal, immune, neurons, glial cells, cancer cells, and stem cells, suggesting that their presence is more ubiquitous than initially thought (see review in Reference [135]). In vivo, TNT-like protrusions called cytonemes have been observed in the imaginal disc development of *Drosophila* [136,137] and in the midgut of the *Anopheles* malaria vector prior to the fertilization of *Plasmodium* gametes [138]. Only recently have other examples of TNT-like structures observed in tissues been reported in malignant tumors dissected from human cancer patients [134,139,140,141,142], in leukemic cells obtained from bone marrow aspirates of pediatric patients [143], and in cardiac myocytes and non-myocyte cells in heart damage [144]. Moreover, an impressive in vivo demonstration of the aforementioned TNT-like structures called TMs has been reported in malignant gliomas, providing even stronger support for a potentially important role of direct intercellular communication by TNTs and GJs in tumor development and progression [21,145]. Ultimately, a central question is: what secondary messengers or organelles are transmitted by GJs, HCs, and TNTs? Furthermore, the mechanism of cell-to-cell recognition remains unknown. Most TNTs are form between stem cells and the target cells. There are not TNTs between cells that do not support carcinogenesis. For example, in HIV, HIV-infected cells only form TNTs with uninfected cells that support HIV replication and cell-to-cell spread. The advantage of TNTs over soluble communication systems is that they are able to transport both small molecules and organelles, such as mitochondria, from cancer cells to adjacent non-cancerous cells without an extracellular component [15].

Cxs, specifically Cx43, are expressed in mitochondria [145,146,147,148], probably as HCs, and function to alter cell metabolism. An important component of cellular metabolism takes place in mitochondria through oxidative phosphorylation (OXPHOS). In the mitochondrial matrix, the Krebs cycle or the tricarboxylic acid cycle (TCA) occurs, transforming pyruvate into energy using electron carriers (NADH and FADH_2_), which subsequently enters the electron transport chain (ETC) where the proton gradient generated by complexes I, III, and IV drives the phosphorylation of ADP to ATP. Thus, the exchange of mitochondria or mitochondrial products affects the metabolism of the target cell, including adaptation to low O_2_ concentration and energy production as well as resistance to apoptosis. Importantly, we have experimentally determined that all of these factors can be transmitted between connected cells via TNTs and GJs or released to the extracellular space via the opening of HCs [15,129,130,149,150]. These findings set the stage for an in-depth investigation to identify therapeutic agents that can effectively and selectively target TNTs and/or GJs in order to prevent this intercellular transfer of mitochondria to thus prevent the spread of the original pathology (e.g., cancer or infection).

In agreement with this idea, our data obtained while studying HIV reservoirs and brain cancer demonstrated that latent HIV-infected or cancerous cells become highly dependent on glutamine/glutamate to produce energy as well as to support TNT formation [151,152,153]. Therefore, the transfer of dysfunctional mitochondria or their metabolites from HIV infected or cancer cells to healthy surrounding cells via GJs or TNTs could alter the proliferation, differentiation, and response to stress (e.g., oxygen and nutrient deprivation) in surrounding areas by TNT dependent mechanism. Furthermore, dysfunctional mitochondria and their products are the major producers of cellular ROS, which can damage key components of cells, including lipids, nucleic acids, and proteins, to spread further carcinogenesis [154,155]. Mitochondrial ROS influence homeostatic signaling pathways to control cell proliferation and differentiation and to contribute to adaptive stress signaling pathways, such as hypoxia, which is a key feature in cancer development [155,156]. Further, ROS produced by complexes I, II, and III have been shown to affect molecular signaling [157]. Complexes I and III produce ROS in the mitochondrial matrix, and complex III releases ROS to both sides of the mitochondrial inner membrane [158]. Another major source of ROS is the NADPH oxidases that catalyze the production of superoxide from O_2_ to NADPH and are Ca^2+^-dependent. It has been proposed that cell death is driven by ROS-dependent signaling pathways [159]. Thus, the direct transfer of these altered mitochondria or derived metabolic products is expected to significantly alter the metabolism and activation status of the target cells, as already observed in different areas of the same tumor-generating heterogeneous differences in tumor growth kinetics [160].

Also, Cx dephosphorylation and the effect of ROS directly on molecules have been suggested to be potential molecular mechanisms that could induce HC opening, resulting in the release of cell survival signal mediators (prostaglandin E_2_ (PGE_2_), ATP, NAD^+^, glutamate) to the extracellular compartment, in addition to an influx of Na^+^, Ca^2+^, and ROS, imbalances in the cellular ionic concentrations, and alterations in cell volume regulation [161]. Most of these products are released by the opening of Cxs and pannexin channels and have significant effects in carcinogenesis [15].

In aerobic glycolysis, tumor cells are also dependent on the glutamine pathway, which provides precursors that are required to increase the proliferation of cells. Glutamine is the most abundant free amino acid in the human blood (400–700 μM) [68,162]. Glutamine is also involved in several metabolic pathways including fatty acid oxidation, the TCA, and the ETC and respiration. TCA acquires particular relevance in cancer principally because of the role of glutamine, which is transformed into glutamate by the enzyme glutaminase. When glutamate is converted into KG by glutamate dehydrogenase, this process makes glutamine the main carbon source for the synthesis of KG, which is used as a source of energy to produce ATP and 4C units of oxaloacetate [163]. During this metabolic cycle, KG generates isocitrate and citrate (2C units), which are used for fatty acid synthesis. Glutamine also serves as a nitrogen source: glutaminase releases a α-amino group, which is used to synthesize nucleotides, asparagine, purines, pyrimidines, nicotinamide adenine dinucleotide (NAD), and glucosamine [68]. Thus, the roles of glutamine and KG are essential in cancer not only for energy production, but also for cell division and proliferation. Under hypoxic conditions, glutamine has been associated with the activation of the oncogenes Ras and Myc, where the former transduces signals to induce proliferation, including the metabolic switch, and the latter is involved in glucose metabolism, as well as nucleotide, lipid, amino acid, and protein synthesis [164]. When these oncogenes are activated simultaneously, the tumor suppressor p53 function becomes compromised [36], which in turn increases the activity of the glutaminolytic pathway, enhancing the ATP and lactate production in cancer cells to promote survival and proliferation. All of these alterations critically contribute to tumor growth. Furthermore, glutamate is the most abundant neurotransmitter in the brain. Thus, in glioblastoma, the use of glutamine/glutamate to generate energy and survive is mostly unlimited and could explain why these tumors are so aggressive. Notably, all of these metabolites are transmitted by GJs, HCs, and TNTs into communicated cells, adding a new dimension to the problem.

As indicated above, under hypoxic conditions glutamine has been associated with the activation of the oncogenes Ras and Myc [164]. Interestingly, Ras reduces the expression of connexin and decreases the levels of membrane-associated Cx43 plaques [165]. Also, the hypophosphorylation of Cx43 was found in normal rat liver epithelial cells compared with cells neoplastically transformed by Myc/Ras [165]. Furthermore, the Cx43 carboxyl terminal group can also regulate cellular proliferation in breast cancer, where p53 exhibits decreased expression in the Cx43 downregulated samples [166]. Additionally, in hepatocellular carcinoma (HCC) tissues, Cx32 regulates the metastasis and proliferation of the tumor [167]. In vitro assays revealed that Cx32 directly enhances the acetylation and transcriptional activity of p53, thus upregulating the expression of the tumor metastasis suppressor protein KAI1/CD82, which is a p53 target gene. Furthermore, Cx32 negatively regulates Akt phosphorylation and cyclin D1 expression, thereby inhibiting the proliferation of HCC cells [167]. It has also been shown that the treatment of rats and mice with the peroxisome proliferator WY-14,643 is associated with an increase in the expression of peroxisomal enzymes required for catalyzing the β-oxidation of fatty acids and of microsomal enzymes catalyzing the ω-oxidation of long-chain polyunsaturated fatty acids. These changes in lipid metabolism show accelerated tumorigenesis in a Cx32-dependent manner [168,169]. In addition, in glioblastoma, it has been concluded that GJs and perhaps HCs promote tumor survival [170,171], and that functional channels promote metastasis [172]. Furthermore, TNT formation has been associated with the activation of all of these pathways [132,173,174,175], suggesting that Cxs expression under cancer conditions and TNT formation may be linked.

## 6. Metabolism of Aggressive Glioblastoma

Glioblastomas are the most aggressive, heterogeneous, and treatment-resistant forms of primary brain cancers. Currently, very few treatment options are available for GB cancers [176,177]. The median five-year survival rate for GB patients over 45 years old is <10% [178]. Recurrence is often a major issue in GB tumors, where residual cancer cells can cause the disease to return within the original tissue site even after concurrent temozolomide (TMZ) and radiation therapy [177]. The recent detection of intercellular TMs is entering the discussion as an additional identifiable characteristic of GBs that may elucidate the interaction of tumor cells with their microenvironment and explain their aggressive clinical behavior [21]. The full extent of the function of these conduits is actively being investigated, but their morphology and functionality are different from neuronal connections found in healthy brain cells [21]. These differences may be important for the exchange of cancer-causing materials, which may explain why GB cancers can proliferate uncontrollably, destroying the surrounding brain tissue, causing severe neurological damage, and rendering any surgical intervention ineffective.

**Figure 3:**
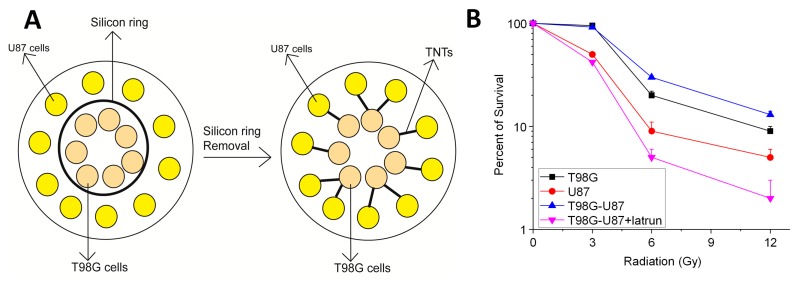
TNTs enable the transfer of protective agents to cancer cells that are susceptible to radiation and chemical treatment. (**A**) Using a silicon coculture system as described, T98G (glioblastoma, resistant to radiation and temozolomide (TMZ) treatment) and U87MG cells (glioblastoma, sensible to radiation and TMZ treatment) were cocultured to examine survival. Upon removal of the silicon barrier, both cell types could form TNTs and communicate with each other. (**B**) Quantification of the survival of T98G and U87 cells alone (back line and red line, respectively) and cocultured U87 cells (blue line). In this case, T98G cells are more resistant to radiation than U87 cells (0 to 12 Gy). However, upon coculturing and the subsequent formation of TNTs, T98G cells transfer the radiation resistance to U87 cells (blue line). The transfer of resistance was dependent on TNT formation, because the use of latrunculin (latrun), a TNT blocker, prevented the transfer of resistance into U87 cells (pink line). Thus, the TNT formation transfers a protective agent against radiation treatment from T98G to U87 cells. Thus, TNTs are essential to spreading chemical and radiation resistance into surrounding cells.

There is growing evidence that TNT-like signaling occurs in cancer and is more common than previously thought [15,179,180]. TNTs are involved in “crosstalk” between cancer stem cells (GB primary cells) and their microenvironment (mesenchymal cells) [129] via GJs and HCs (see Figure 3). For example, as described in Figure 3, TNTs mediate the transfer of metabolites and/or organelles that provide resistance to radiation and TMZ treatment. To perform these studies, two GB cell lines were used: T98G (radioresistant) and U87MG (radiosensitive). As shown in Figure 3A, the cells were co-cultured, but separated by a silicon barrier to prevent contact. Upon removal of the silicon barrier, they readily established TNT connections. Both cell lines were treated with various doses of radiation (from 0 to 12 Gy), and cell survival was assessed after 72 h. As expected, we found that pure cultures of U87 cells were more sensitive to radiation than T98G (Figure 3B black line). Notably, when U87 and T98G were cocultured, the formation of TNTs transmitted a protective factor(s) against radiation from T98G to U87 cells, increasing their survival (Figure 3B blue line). When both cell lines were cocultured in the presence of 1 nM of latrunculin (an actin destabilizing agent that is commonly used to prevent the formation of TNTs in vitro), the transfer of the protective factors by TNTs was inhibited, and the survival of U87 cells was decreased following exposure to radiation (Figure 3B pink line). These results indicate that TNTs are able to transfer a protective factor from radiation-resistant to radiation-susceptible cells, altering the phenotype of the latter to make them resistant to radiation.

## 7. Connexin Channels: Novel Roles in Cancer

All of the aspects described above are controlled and regulated by GJs and HCs. However, several great review articles describe in detail the well-accepted participation of these channels in metabolism, cancer, synapses, and the recirculation of neurotransmitters and energy molecules [30,33,39,131,181,182,183,184]. Thus, we will now focus on the novel aspects of these channels in cancer pathogenesis.

To date, most reports indicate that connexin expression and GJ communication is reduced or lost in cancer cells and remains expressed in a localized manner in many types of cancer [30,185,186,187]. For example, local GJ communication regulates CD90 (Thy-1) expression by cancer cells, especially leukemia, supporting their role in the dedifferentiation of cells into a fetal stage [188]. MicroRNAs (miRNAs) that could affect the expression of several chemokines also can be transferred by GJs present in tumors, resulting in an alteration in the migration of immune and tumor cells [189]. Furthermore, the transfer of miRNA from glioma cells to healthy astrocytes has been shown to enhance the pro-invasive nature of gliomas [190]. In contrast, the transfer of miR124-3p had anti-proliferative effects, demonstrating a bystander communication between tumor-tumor and tumor-healthy cells [191]. After these initial reports, several groups suggested that several forms of miRNA associated with chemoresistance, and potentially other kinds of genetic material, could provide survival and chemotherapy resistance by a mechanism mediated by GJs and TNTs [140,192,193,194,195].

With respect to HCs, initially these channels were associated with vascular disruption and hemorrhage inside tumors [196]. The opening of Cx43-containing HCs has been associated with the suppression of breast cancer proliferation and metastasis [197]. In several cancers, the opening of HCs results in the release of significant amounts of PGE_2_, which regulates immune cell activation and protects lymphoblastic leukemia cells from other cells [198,199]. These results indicate that the opening of HCs also promotes the survival and metastasis of cancer cells. However, the role of HCs in the pathogenesis of cancer needs to be further examined.

Several recent reports provide strong evidence supporting the expression and role of TNTs as being similar to mechanisms in treatment-resistant cancers such as gliomas, leukemia and ovarian cancer [129,200]. In gliomas, ultra-long functional TNT-like membrane protrusions (called tumor microtubules or TMs) [141,143,201,202] were observed in mice [21,128,129,203] to form a distinct multi-cellular network over time. These TMs were functional and mediated the transfer of nuclear material from cancer cells to neighboring brain cells. In addition, it has been reported that the local expression of Cx43 (and perhaps other Cxs) also amplifies tumor resistance by modulating mitochondrial function [204], but the mechanisms involved are still unclear. Therefore, we propose that better understanding the biology, morphology, and function of TNTs and their association with GJs and HCs will be important for generating new opportunities for pharmacological intervention and therapeutic strategies against brain cancer and related pathologies.

As an alternative to channel blocking, a more revolutionary approach could be to exploit and “hijack” the intercellular TNT network to deliver local or cerebrospinal fluid (CSF)-injected toxic drugs to distant tumor cells. Indeed, several groups demonstrated that transfection of the tumor with Cx43 enhanced the effects of genetic therapies. This was accomplished by infecting glioma cells with the herpes simplex virus thymidine kinase (HSVtk) gene that can result in cell death after treatment with ganciclovir (GCV), a nucleoside analog [205,206,207,208,209,210,211]. GCV is phosphorylated by HSVtk into a monophosphate form and subsequently to GCV-triphosphate by endogenous kinases. It is then incorporated into the DNA of the target cell, leading to strand breaks and resulting in cell death. Interestingly, neighboring cells coupled by GJs also die, although these cells do not express the enzyme [150]. This phenomenon is believed to be caused by a bystander effect mediated by the GJ-mediated transfer of toxic GCV metabolites from the cell infected with HSVtk to uninfected neighbor cells [212]. Cx43 transfection into tumor cells was shown to result in functional coupling and in the enhancement of the bystander effect in vivo [206,213,214] and in vitro [205,206,207,208,209,213,214,215,216]. Furthermore, the bystander effect can also occur via TNTs [150]. Ady et al. used an engineered herpes simplex virus (HSV) expressing green fluorescent protein (GFP) to visualize the intercellular transfer of both GFP and the virus from infected to non-infected cells via TNTs [150]. Cells were further co-cultured but separated using a trans-well membrane to prevent GJ connections; the addition of GCV to the virus-infected population nonetheless still resulted in amplified cell toxicity via the bystander effect, identifying TNTs as a novel additional mechanism by which this effect can take place and establishing additional common functionality with GJs. Several groups continue to optimize the potential of the bystander effect for therapeutic treatment of solid tumors in conjunction with GJ and TNT communication to spread toxicity into neighboring cells.

## 8. Future Directions and Conclusions

We believe that localized GJs, HCs, and TNTs all play key roles in carcinogenesis and cancer spread. Blocking these communication systems could therefore prevent cancer progression by compromising critical underlying mechanisms of intercellular communication. The current paradigms regarding the functions of local GJs, HCs, and TNTs are that these channels participate in a wide range of processes, including but not limited to the targeted self-renewal of cancer stem cells, differentiation, metabolism, proliferation, and metastasis.
